# Supplementary material for: Sequence-Dependent Orientational Coupling and Electrostatic Attraction in Cation-Mediated DNA–DNA Interactions
Source: J Chem Theory Comput. 2023 Sep 20;19(19):6827–38. doi: 10.1021/acs.jctc.3c00520 (PMC10569048; doi:10.1021/acs.jctc.3c00520)
Supplement: Supplementary file 1 — ct3c00520_si_001.pdf [file ct3c00520_si_001.pdf]

Supporting information for Sequence-dependent orientational coupling and electrostatic attraction in cation-mediated DNA-DNA interactions

Weiwei He,[†,‡] Xiangyun Qiu,[*,¶] and Serdal Kirmizialtin[*,†,‡]

†*Chemistry Program, Science Division, New York University Abu Dhabi, Abu Dhabi, 129188, United Arab Emirates*

‡*Department of Chemistry, New York University, New York, NY 10012, United States*

¶*Department of Physics, George Washington University, Washington DC 20052, United States*

E-mail: xqiu@gwu.edu; serdal@nyu.edu

Molecular modeling of DNA duplexes

The initial structures of both ATDNA and MixDNA constructs were created using Nucleic Acid Builder (NAB),[1] assuming B-form geometry. ATDNA refers to a 20bp duplex consisting of two homopolymeric chains, dA20 and dT20, while MixDNA represents a 20bp duplex with a mixed sequence of GCA TCT GGGC TATA AAA GGG and its complementing sequence. These structures were duplicated to create two parallel arrays, which were then placed in a rectangular cell with the long axis aligned to the z-coordinate. This resulted in an initial simulation box size of $11.8nm \times 11.8nm \times 6.8nm$. Periodic boundary conditions (PBC) were applied to extend the DNA to infinite length. To solvate the DNA, we used explicit water and ions. We studied the ATDNA and MixDNA systems in pure Mg^{2+}, as well as ATDNA in two different mixed salt conditions. Table S1 summarizes each simulation setup and salt conditions.

Table S1

Simulation system	ATDNA Mg^{2+}	MixDNA Mg^{2+}	ATDNA Mg^{2+}/Na^{+}	ATDNA Mg^{2+}/K^{+}	ATDNA Mg^{2+}/Na^{+} /$Spermine^{4+}$
Salt condition	52.5mM Mg^{2+}	52.3mM Mg^{2+}	15.2mM Mg^{2+} 119.8mM Na^{+}	12.5mM Mg^{2+} 115.6mM K^{+}	16.6mM Mg^{2+} 86.5mM Na^{+} 0.21mM $Spermine^{4+}$

General MD simulation set up

All simulations were carried out using GROMACS 2018.5[2] suite of programs. We used amber99sb_parmbsc0[3] force field for DNA, NBFIX[4] parameters for Mg, Na, K and Cl, and TIP3P[5] for water molecules. For spermine parameters we adopted NBFIX corrections[6] while we computed partial charges using RESP procedure.[7] As a benchmark, we compared our free energy minima with Fig.1c of Ref[8]

Once the simulation system is set up we employed a 5000-step energy minimization to remove any bad contacts resulting from the random placement of water and ions. The minimized simulation set ups later used for MD simulations.

Next, an equilibrium procedure involving volume and solvent was performed on the minimized structures. Specifically, we carried out two nanoseconds of constrained MD at NPT to adjust the simulation volume, followed by 200-ns of constrained MD at canonical ensemble to equilibrate the water and ions around the restrained DNA molecules.

The equations of motion were integrated using the Leap-Frog scheme with a 2-fs time step. Particle mesh Ewald (PME) summation method was used to treat electrostatics, with a grid spacing of 0.12 nm and an interpolation of order 4. Non-bonded interactions and neighbor searches were cutoff at 11 Å, and the list was updated every 40 steps. The covalent bonds of water and nucleic acid were constrained using SETTLE[9] and LINCS[10] algorithms, respectively. We used Berendsen thermostat[11] and Parrinello-Rahman barostat[12] for NPT simulations and velocity scaling[13] for NVT simulations. During the equilibration, all heavy atoms of DNA were restrained with a stiffness constant of 1000 kJ mol^{-1} nm^{-2}, while water and ions were allowed to move freely. The output coordinate of the NVT simulation was used as the starting point for the following Well-tempered metadynamics simulations.

Well-tempered metadynamics simulations

Well-tempered Metadynamics (WTMD)[14] was employed to extensively sample the conformational space of two parallel DNA pairs. To describe the dynamics, we employed two collective variables (CVs): inter-helical distance (d) and inter-helical rotation (θ) (Fig. 1a-b). During the WTMD runs, we constrained the orientation of the center helix (H1) by applying a harmonic restraint with a stiffness constant of 1000 $kJ \cdot mol^{-1} \cdot nm^{-2}$. Gaussians were initialized with a magnitude of W=0.6 kJ/mol and deposited every 1 ps with a gradually decreasing bias factor of 6.0. The widths of the Gaussians were set to $\sigma_d = 0.1$ nm for d and $\sigma_\theta = 0.2$ rad for θ, respectively. The heights of the deposited Gaussians and

the collective variables were monitored during the simulations (Fig. 1c). Following,[15,16] the decay of the heights of the deposited Gaussians to < 0.005 kJ/mol was used as a threshold to assess the convergence. In addition, the convergence of each simulation was assessed by performing block analysis (Fig. S1).

Data Analysis

We monitored the position of each cation in time. This data is then used to compute the average density and charge profiles, and entropy.

Computing the entropy of Mg^{2+} and water using 2PT method

We used the DoSPT program[17] to apply the 2PT method and estimate the entropic contribution of Mg^{2+} with its hexa-hydrated shell (i.e., $Mg(H_2O)_6{}^{2+}$). We studied cation entropy in the condensed state (with inter-helical distances of ∼2.8 nm) and in the free state (with inter-helical distances of ∼4.0 nm). To do this we first generate a pool of structures sampling each state. For that we performed 150-ns-long MD simulations at 300 K, with the inter-helical distances restrained to the states defined. We selected a conformation every 5 ns (resulting in 30 starting points). From each conformation selected we conducted a 120-ps-long NVT simulation with $\delta t = 1$ fs resolution and we recorded the forces, velocities, and coordinates in 4 fs time intervals. The saved trajectories were then used in DoSPT program to estimate the entropy. The convergence of the entropy calculation was assessed using trajectories of varying lengths (80, 100, 120, and 150 ps), as shown in Fig. S2. The error bar was computed as the standard deviation by taking the average of these 30 independent DoSPT runs, it accounts for any variability in the results that could arise from small differences in the initial conditions or other factors.

Similarly, we estimate the entropy of solvent water using the same approach. The 2PT calculation of water entropy is known to converge quickly, short (∼10 ps) MD simulations provide very accurate values of energy and entropy,[18] we run 20ps in this study. We evaluate the bound water defined by the R1 hydration shell (Fig. 1c) and bulk water, respectively. A ∼50 mM $MgCl_2$ solution was simulated for 50ns as pure-solvent system in order to compute the entropy of water in bulk (S_{bulk}). As for the entropy of bound water, we pick the same starting points applied in entropy calculation of Mg^{2+}. This time only the free state is considered, the water molecules within 3.5Å (R1 shell) of DNAs' surface were used to perform DoSPT calculations (S_{bound}). The entropic contribution of bound water released to the bulk (Δn) occurred during free-state-to-condensed-state transition is was then expressed as $\Delta S_{water} = \Delta n(S_{bulk} - S_{bound})$ (Table 1).

Computing the entropy of DNA duplexes using multiscale cell correlation method

Using the CodeEntropy program, which implements MCC theory,[19,20] we calculated the macromolecular entropy of DNAs. Similar to the cation entropy analysis, we utilized the two end-state trajectories. We performed 100-ns-long MD simulations at canonical ensemble

with a temperature of 300K and restrained the inter-helical distance at the desired values to record the forces every 10 ps. These forces were loaded into the MCC program to estimate the macromolecular entropy. To estimate the error, we divided the trajectory into two equal parts. In our study, we found that the topographical entropy S_M^{topo} was small due to the relatively stable backbone structure and was hence neglected.

Cation density profiles and water structuring

We compute the average cation density profiles and tetrahedral order parameters from the 150 ns long MD trajectories described above. For density analysis, we investigate (e.g. pure Mg^{2+}, Mg^{2+}/Na^{+}, Mg^{2+}/K^{+} and Mg^{2+}/Na^{+}/Spermine^{4+}) at the condensed and free states. Due to linear charge distribution of Spermine^{4+}, we divided the molecule into 4 parts according to the position of its four nitrogen atoms during the calculation (Fig. 7c, bottom).

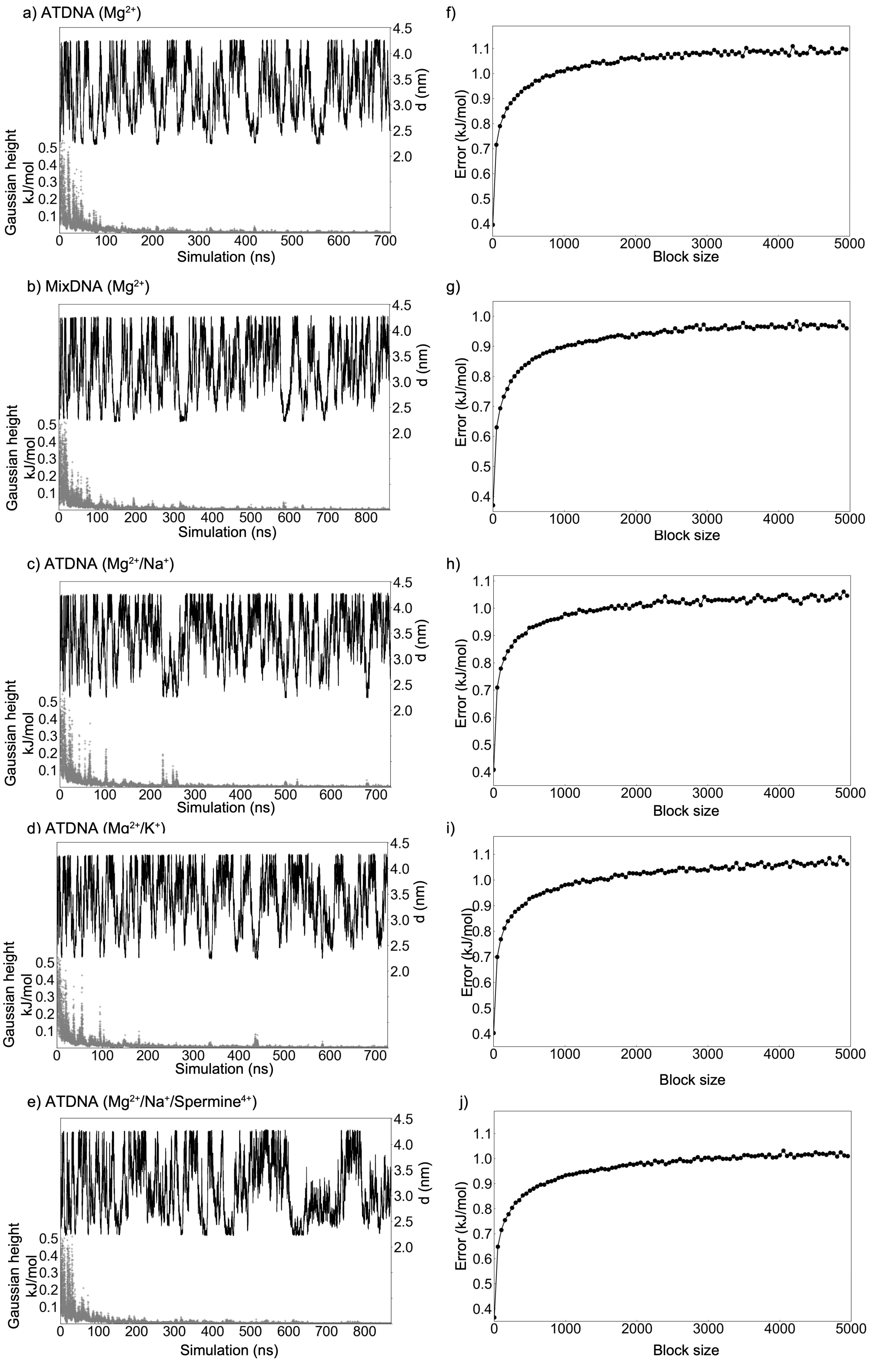

Figure S1: Time evolution of the collective variable d (black) and Gaussian hills (gray) during metadynamics simulations and block analysis. a)/f) ATDNA in Mg^{2+}, b)/g) MixDNA in Mg^{2+}, c)/h) ATDNA in Mg^{2+}/Na^{+}, d)/i) ATDNA in Mg^{2+}/K^{+} and e)/j) ATDNA in $Mg^{2+}/Na^{+}/Spermine^{4+}$.

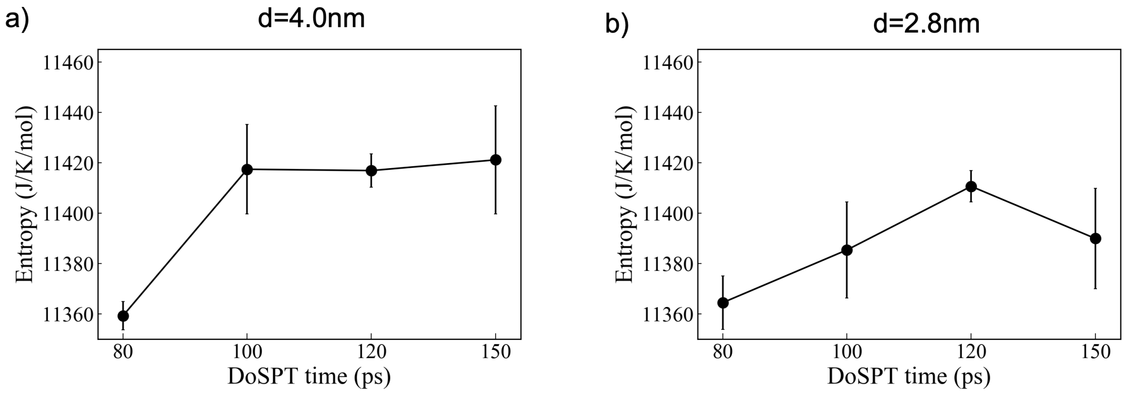

Figure S2: Convergence of the DoSPT entropy calculations for the ATDNA/Mg^{2+} system. The graph illustrates the convergence behavior of the DoSPT calculations as a function of simulation time.

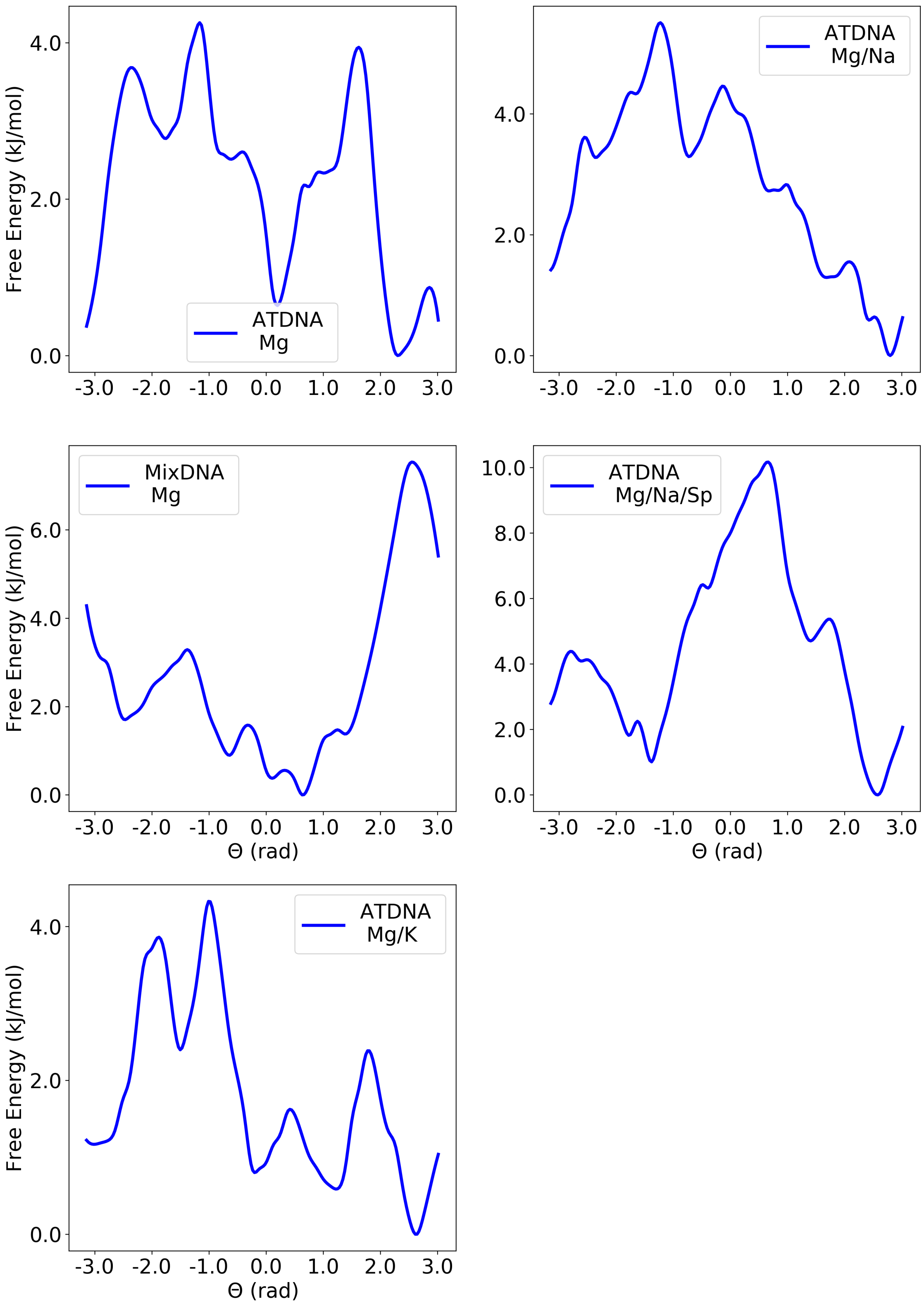

Figure S3: Free energy profiles projected on the collective variable θ.

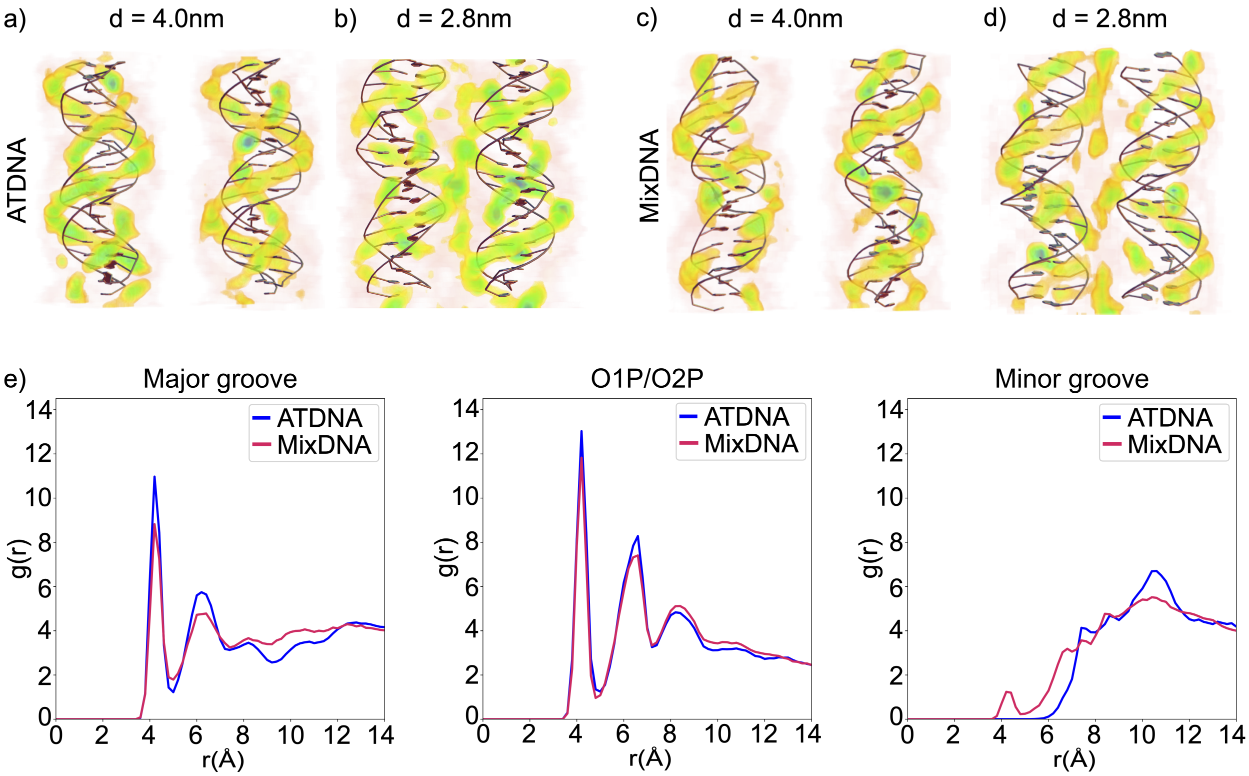

Figure S4: The change of average Mg^{2+} ion distribution around the duplexes during the dissociation-to-association transition. We plot the 3D ion density map for a) free state and b) condensed state of ATDNA and the c-d) two sates for MixDNA. e) Radial distribution function of Mg^{2+} ions from the major group atoms (left), phosphate backbone (central) and minor groove atoms (right) for ATDNA (blue solid) and MixDNA (green solid) in pure Mg^{2+} solution, respectively.

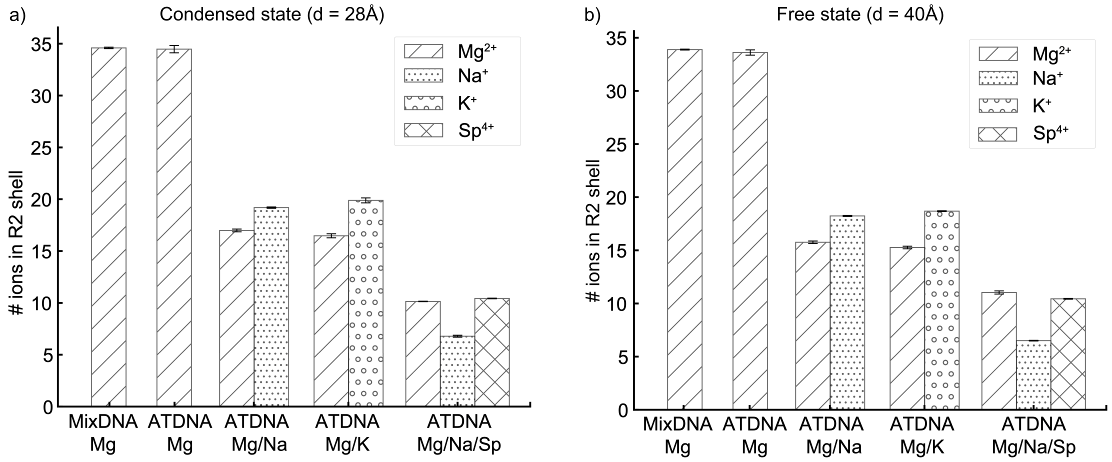

Figure S5: Ion coordination analysis. The number of cations within the R2 shell, defined in Fig. 3, was counted for each case.

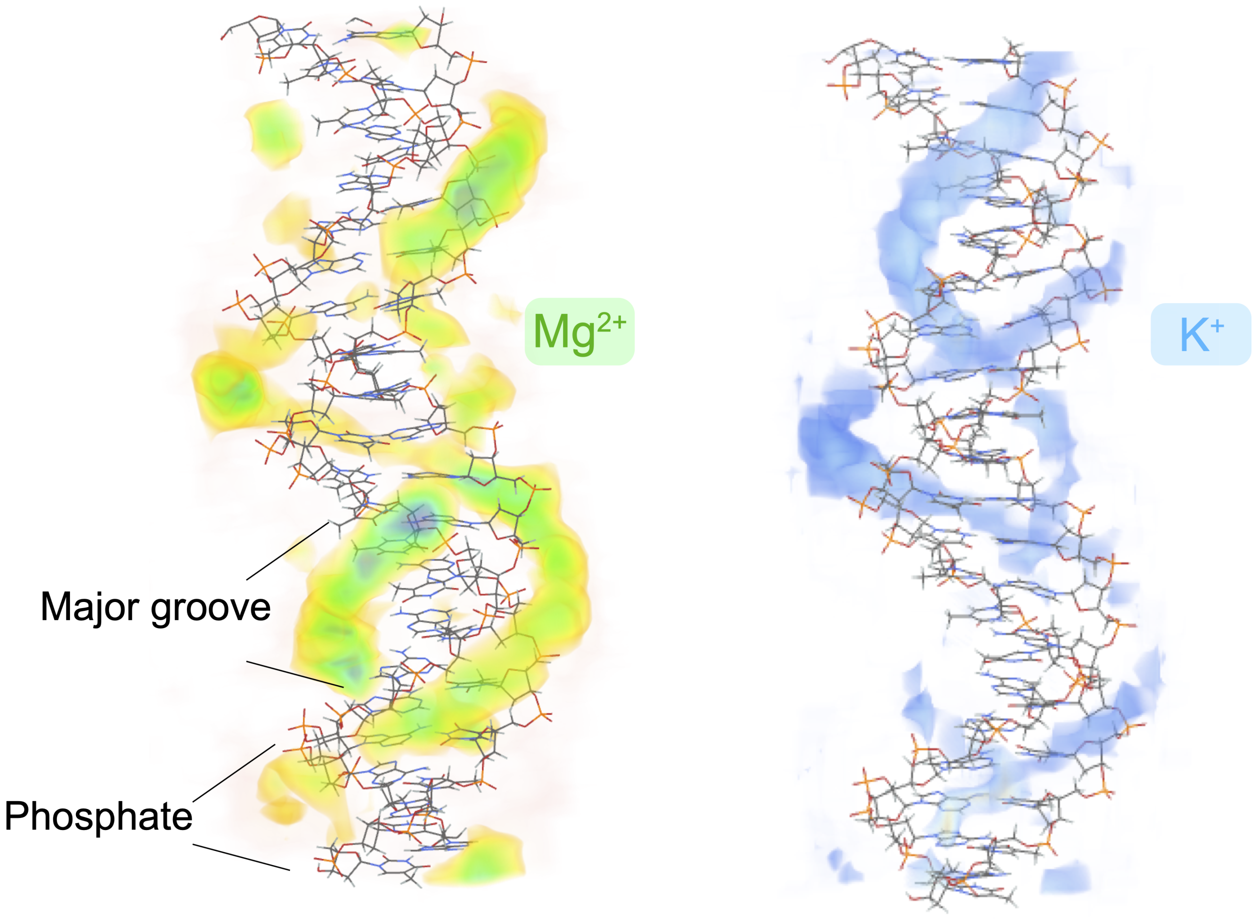

Figure S6: Spatial distribution of cations around the DNA duplex in K-Mg. The 3D density map of cations in mixture of Mg^{2+} (green) and K^{+} (cyan).

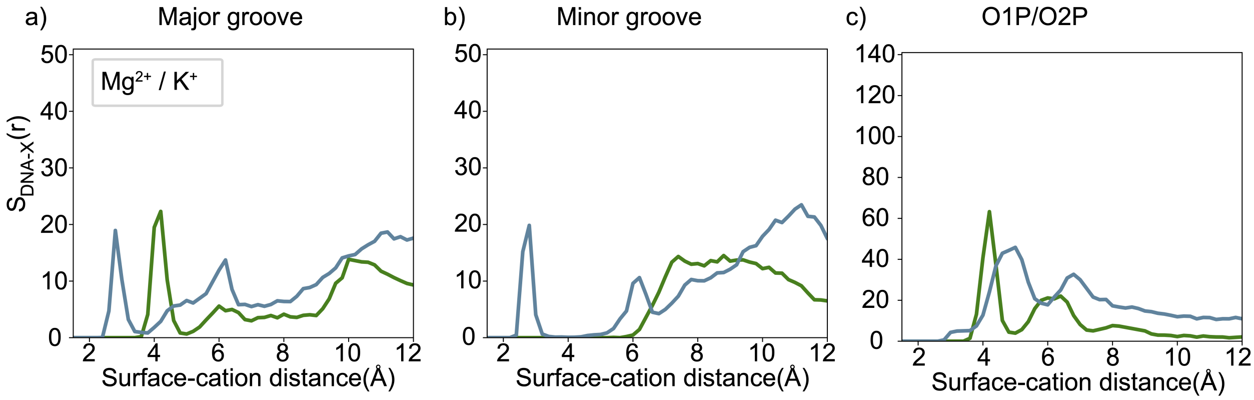

Figure S7: The surface radial distribution function of cations around the DNA duplex in K-Mg. a) major group atoms, b) minor groove atoms, and c) phosphate backbone. Analogous to Fig. 7, the green line represents Mg^{2+} and the Y-axis scale is set to the same as Fig. 7 for comparison.

Movie 1. Molecular simulation of ATDNA in Mg^{2+}

Movie 2. Molecular simulation of ATDNA in a mixture of Mg^{2+}, Na^{+} and Sp^{4+}

Spermine (Sp) parameters in itp format:
; GROMACS topology file for spermine (force field Amber_Cornell_ext.params+)
[moleculetype]
; Name nrexcl
Sp 3
[atoms]
; nr type resnr residu atom cgnr charge mass typeB chargeB massB
1 NP 1 Sp NP1 1 -0.009484 14.0067 ; qtot 0
2 CT2 1 Sp CT21 2 0.000587 12.0107 ; qtot 0
3 CT2 1 Sp CT22 3 0.025206 12.0107 ; qtot 0
4 CT2 1 Sp CT23 4 0.025909 12.0107 ; qtot 0
5 CT2 1 Sp CT24 5 0.002024 12.0107 ; qtot 0
6 NP 1 Sp NP2 6 -0.018800 14.0067 ; qtot 0
7 HP 1 Sp HP1 7 0.089567 1.00794 ; qtot 0
8 HP 1 Sp HP2 8 0.089893 1.00794 ; qtot 0
9 HC 1 Sp HC1 9 0.032275 1.00794 ; qtot 0
10 HC 1 Sp HC2 10 0.032122 1.00794 ; qtot 0
11 HC 1 Sp HC3 11 0.031456 1.00794 ; qtot 0
12 HC 1 Sp HC4 12 0.032131 1.00794 ; qtot 0
13 HP 1 Sp HP3 13 0.090312 1.00794 ; qtot 0
14 HP 1 Sp HP4 14 0.089498 1.00794 ; qtot 0
15 H 1 Sp H1 15 0.243679 1.00794 ; qtot 0
16 CT2 1 Sp CT25 16 0.046240 12.0107 ; qtot 0
17 CT2 1 Sp CT26 17 -0.130823 12.0107 ; qtot 0
18 CT2 1 Sp CT27 18 0.255010 12.0107 ; qtot 0
19 NH3 1 Sp NT1 19 -0.361267 14.0067 ; qtot 0
20 CT2 1 Sp CT28 20 0.043292 12.0107 ; qtot 0
21 CT2 1 Sp CT29 21 -0.139168 12.0107 ; qtot 0
22 CT2 1 Sp CT210 22 0.258347 12.0107 ; qtot 0
23 NH3 1 Sp NT2 23 -0.363586 14.0067 ; qtot 0
24 H 1 Sp H2 24 0.246765 1.00794 ; qtot 0
25 HP 1 Sp HP5 25 0.083368 1.00794 ; qtot 0
26 HP 1 Sp HP6 26 0.083630 1.00794 ; qtot 0
27 HC 1 Sp HC5 27 0.073488 1.00794 ; qtot 0
28 HC 1 Sp HC6 28 0.074204 1.00794 ; qtot 0
29 HP 1 Sp HP7 29 0.057130 1.00794 ; qtot 0
30 HP 1 Sp HP8 30 0.057332 1.00794 ; qtot 0
31 HP 1 Sp HP9 31 0.084549 1.00794 ; qtot 0
32 HP 1 Sp HP10 32 0.084643 1.00794 ; qtot 0
33 HC 1 Sp HC7 33 0.076354 1.00794 ; qtot 0
34 HC 1 Sp HC8 34 0.076205 1.00794 ; qtot 0
35 HP 1 Sp HP11 35 0.057112 1.00794 ; qtot 0
36 HP 1 Sp HP12 36 0.057264 1.00794 ; qtot 0
37 H 1 Sp H3 37 0.348424 1.00794 ; qtot 0
38 H 1 Sp H4 38 0.333434 1.00794 ; qtot 0

39 H 1 Sp H5 39 0.334523 1.00794 ; qtot 0
40 H 1 Sp H6 40 0.334452 1.00794 ; qtot 0
41 H 1 Sp H7 41 0.348168 1.00794 ; qtot 0
42 H 1 Sp H8 42 0.333912 1.00794 ; qtot 0
43 H 1 Sp H9 43 0.244132 1.00794 ; qtot 0
44 H 1 Sp H10 44 0.246491 1.00794 ; qtot 0
[bonds]
; ai aj funct
2 1 1 ;
3 2 1 ; CT CT
4 3 1 ; CT CT
5 4 1 ; CT CT
6 5 1 ; N4 CT
7 2 1 ; HC CT
8 2 1 ; HC CT
9 3 1 ; HC CT
10 3 1 ; HC CT
11 4 1 ; HC CT
12 4 1 ; HC CT
13 5 1 ; HC CT
14 5 1 ; HC CT
15 1 1 ; H N4
16 6 1 ; CT N4
17 16 1 ; CT CT
18 17 1 ; CT CT
19 18 1 ; N4 CT
20 1 1 ; CT N4
21 20 1 ; CT CT
22 21 1 ; CT CT
23 22 1 ; N4 CT
24 6 1 ; H N4
25 16 1 ; HC CT
26 16 1 ; HC CT
27 17 1 ; HC CT
28 17 1 ; HC CT
29 18 1 ; HC CT
30 18 1 ; HC CT
31 20 1 ; HC CT
32 20 1 ; HC CT
33 21 1 ; HC CT
34 21 1 ; HC CT
35 22 1 ; HC CT
36 22 1 ; HC CT
37 19 1 ; H N4
38 19 1 ; H N4

39 23 1 ; H N4
40 23 1 ; H N4
41 23 1 ; H N4
42 19 1 ; H N4
43 1 1 ; H N4
44 6 1 ; H N4
[pairs]
; ai aj funct
3 15 1 ;CT CT N4 H
3 20 1 ;CT CT N4 CT
3 43 1 ;CT CT N4 H
7 15 1 ;HC CT N4 H
7 20 1 ;HC CT N4 CT
7 43 1 ;HC CT N4 H
8 15 1 ;HC CT N4 H
8 20 1 ;HC CT N4 CT
8 43 1 ;HC CT N4 H
4 1 1 ;CT CT CT N4
4 7 1 ;CT CT CT HC
4 8 1 ;CT CT CT HC
9 1 1 ;HC CT CT N4
9 7 1 ;HC CT CT HC
9 8 1 ;HC CT CT HC
10 1 1 ;HC CT CT N4
10 7 1 ;HC CT CT HC
10 8 1 ;HC CT CT HC
5 2 1 ;CT CT CT CT
5 9 1 ;CT CT CT HC
5 10 1 ;CT CT CT HC
11 2 1 ;CT CT CT HC
11 9 1 ;HC CT CT HC
11 10 1 ;HC CT CT HC
12 2 1 ;CT CT CT HC
12 9 1 ;HC CT CT HC
12 10 1 ;HC CT CT HC
6 3 1 ;CT CT CT N4
6 11 1 ;HC CT CT N4
6 12 1 ;HC CT CT N4
13 3 1 ;CT CT CT HC
13 11 1 ;HC CT CT HC
13 12 1 ;HC CT CT HC
14 3 1 ;CT CT CT HC
14 11 1 ;HC CT CT HC
14 12 1 ;HC CT CT HC
16 4 1 ;CT CT N4 CT

16 13 1 ;HC CT N4 CT
16 14 1 ;HC CT N4 CT
24 4 1 ;CT CT N4 H
24 13 1 ;HC CT N4 H
24 14 1 ;HC CT N4 H
44 4 1 ;CT CT N4 H
44 13 1 ;HC CT N4 H
44 14 1 ;HC CT N4 H
17 5 1 ;CT CT N4 CT
17 24 1 ;CT CT N4 H
17 44 1 ;CT CT N4 H
25 5 1 ;HC CT N4 CT
25 24 1 ;HC CT N4 H
25 44 1 ;HC CT N4 H
26 5 1 ;HC CT N4 CT
26 24 1 ;HC CT N4 H
26 44 1 ;HC CT N4 H
18 6 1 ;CT CT CT N4
18 25 1 ;CT CT CT HC
18 26 1 ;CT CT CT HC
27 6 1 ;HC CT CT N4
27 25 1 ;HC CT CT HC
27 26 1 ;HC CT CT HC
28 6 1 ;HC CT CT N4
28 25 1 ;HC CT CT HC
28 26 1 ;HC CT CT HC
19 16 1 ;CT CT CT N4
19 27 1 ;HC CT CT N4
19 28 1 ;HC CT CT N4
29 16 1 ;CT CT CT HC
29 27 1 ;HC CT CT HC
29 28 1 ;HC CT CT HC
30 16 1 ;CT CT CT HC
30 27 1 ;HC CT CT HC
30 28 1 ;HC CT CT HC
37 17 1 ;CT CT N4 H
37 29 1 ;HC CT N4 H
37 30 1 ;HC CT N4 H
38 17 1 ;CT CT N4 H
38 29 1 ;HC CT N4 H
38 30 1 ;HC CT N4 H
42 17 1 ;CT CT N4 H
42 29 1 ;HC CT N4 H
42 30 1 ;HC CT N4 H
21 2 1 ;CT CT N4 CT

21 15 1 ;CT CT N4 H
21 43 1 ;CT CT N4 H
31 2 1 ;HC CT N4 CT
31 15 1 ;HC CT N4 H
31 43 1 ;HC CT N4 H
32 2 1 ;HC CT N4 CT
32 15 1 ;HC CT N4 H
32 43 1 ;HC CT N4 H
22 1 1 ;CT CT CT N4
22 31 1 ;CT CT CT HC
22 32 1 ;CT CT CT HC
33 1 1 ;HC CT CT N4
33 31 1 ;HC CT CT HC
33 32 1 ;HC CT CT HC
34 1 1 ;HC CT CT N4
34 31 1 ;HC CT CT HC
34 32 1 ;HC CT CT HC
23 20 1 ;CT CT CT N4
23 33 1 ;HC CT CT N4
23 34 1 ;HC CT CT N4
35 20 1 ;CT CT CT HC
35 33 1 ;HC CT CT HC
35 34 1 ;HC CT CT HC
36 20 1 ;CT CT CT HC
36 33 1 ;HC CT CT HC
36 34 1 ;HC CT CT HC
39 21 1 ;CT CT N4 H
39 35 1 ;HC CT N4 H
39 36 1 ;HC CT N4 H
40 21 1 ;CT CT N4 H
40 35 1 ;HC CT N4 H
40 36 1 ;HC CT N4 H
41 21 1 ;CT CT N4 H
41 35 1 ;HC CT N4 H
41 36 1 ;HC CT N4 H
[angles]
; ai aj ak funct
2 1 15 5 ; CT2 NP H
2 1 20 5 ; CT2 NP CT2
2 1 43 5 ; CT2 NP H
15 1 20 5 ; H NP H
15 1 43 5 ; H NP H
20 1 43 5 ; CT2 NP H
1 2 3 5 ; NP CT2 CT2
1 2 7 5 ; NP CT2 HP

1 2 8 5 ; NP CT2 HP
3 2 7 5 ; CT2 CT2 HP
3 2 8 5 ; CT2 CT2 HP
7 2 8 5 ; HP CT2 HP
2 3 4 5 ; CT2 CT2 CT2
2 3 9 5 ; CT2 CT2 HC
2 3 10 5 ; CT2 CT2 HC
4 3 9 5 ; CT2 CT2 HC
4 3 10 5 ; CT2 CT2 HC
9 3 10 5 ; HC CT2 HC
3 4 5 5 ; CT2 CT2 CT2
3 4 11 5 ; CT2 CT2 HC
3 4 12 5 ; CT2 CT2 HC
5 4 11 5 ; CT2 CT2 HC
5 4 12 5 ; CT2 CT2 HC
11 4 12 5 ; HC CT2 HC
4 5 6 5 ; CT2 CT2 NP
4 5 13 5 ; CT2 CT2 HP
4 5 14 5 ; CT2 CT2 HP
6 5 13 5 ; NP CT2 HP
6 5 14 5 ; NP CT2 HP
13 5 14 5 ; HP CT2 HP
5 6 16 5 ; CT2 NP CT2
5 6 24 5 ; CT2 NP H
5 6 44 5 ; CT2 NP H
16 6 24 5 ; CT2 NP H
16 6 44 5 ; CT2 NP H
24 6 44 5 ; H NP H
6 16 17 5 ; NP CT2 CT2
6 16 25 5 ; NP CT2 HP
6 16 26 5 ; NP CT2 HP
17 16 25 5 ; CT2 CT2 HP
17 16 26 5 ; CT2 CT2 HP
25 16 26 5 ; HP CT2 HP
16 17 18 5 ; CT2 CT2 CT2
16 17 27 5 ; CT2 CT2 HC
16 17 28 5 ; CT2 CT2 HC
18 17 27 5 ; CT2 CT2 HC
18 17 28 5 ; CT2 CT2 HC
27 17 28 5 ; HC CT2 HC
17 18 19 5 ; CT2 CT2 NH3
17 18 29 5 ; CT2 CT2 HP
17 18 30 5 ; CT2 CT2 HP
19 18 29 5 ; NH3 CT2 HP
19 18 30 5 ; NH3 CT2 HP

29 18 30 5 ; HP CT2 HP
18 19 37 5 ; CT2 NH3 H
18 19 38 5 ; CT2 NH3 H
18 19 42 5 ; CT2 NH3 H
37 19 38 5 ; H NH3 H
37 19 42 5 ; H NH3 H
38 19 42 5 ; H NH3 H
1 20 21 5 ; NP CT2 CT2
1 20 31 5 ; NP CT2 HP
1 20 32 5 ; NP CT2 HP
21 20 31 5 ; CT2 CT2 HP
21 20 32 5 ; CT2 CT2 HP
31 20 32 5 ; HP CT2 HP
20 21 22 5 ; CT2 CT2 CT2
20 21 33 5 ; CT2 CT2 HC
20 21 34 5 ; CT2 CT2 HC
22 21 33 5 ; CT2 CT2 HC
22 21 34 5 ; CT2 CT2 HC
33 21 34 5 ; HC CT2 HC
21 22 23 5 ; CT2 CT2 NH3
21 22 35 5 ; CT2 CT2 HP
21 22 36 5 ; CT2 CT2 HP
23 22 35 5 ; NH3 CT2 HP
23 22 36 5 ; NH3 CT2 HP
35 22 36 5 ; HP CT2 HP
22 23 39 5 ; CT2 NH3 H
22 23 40 5 ; CT2 NH3 H
22 23 41 5 ; CT2 NH3 H
39 23 40 5 ; H NH3 H
39 23 41 5 ; H NH3 H
40 23 41 5 ; H NH3 H
[dihedrals]
; ai aj ak al funct
3 2 1 15 9 ;CT CT N4 H
3 2 1 20 9 ;CT CT N4 CT
3 2 1 43 9 ;CT CT N4 H
7 2 1 15 9 ;HC CT N4 H
7 2 1 20 9 ;HC CT N4 CT
7 2 1 43 9 ;HC CT N4 H
8 2 1 15 9 ;HC CT N4 H
8 2 1 20 9 ;HC CT N4 CT
8 2 1 43 9 ;HC CT N4 H
4 3 2 1 9 ;CT CT CT N4
4 3 2 7 9 ;CT CT CT HC
4 3 2 8 9 ;CT CT CT HC

9 3 2 1 9 ;HC CT CT N4
9 3 2 7 9 ;HC CT CT HC
9 3 2 8 9 ;HC CT CT HC
10 3 2 1 9 ;HC CT CT N4
10 3 2 7 9 ;HC CT CT HC
10 3 2 8 9 ;HC CT CT HC
5 4 3 2 9 ;CT CT CT CT
5 4 3 9 9 ;CT CT CT HC
5 4 3 10 9 ;CT CT CT HC
11 4 3 2 9 ;CT CT CT HC
11 4 3 9 9 ;HC CT CT HC
11 4 3 10 9 ;HC CT CT HC
12 4 3 2 9 ;CT CT CT HC
12 4 3 9 9 ;HC CT CT HC
12 4 3 10 9 ;HC CT CT HC
6 5 4 3 9 ;CT CT CT N4
6 5 4 11 9 ;HC CT CT N4
6 5 4 12 9 ;HC CT CT N4
13 5 4 3 9 ;CT CT CT HC
13 5 4 11 9 ;HC CT CT HC
13 5 4 12 9 ;HC CT CT HC
14 5 4 3 9 ;CT CT CT HC
14 5 4 11 9 ;HC CT CT HC
14 5 4 12 9 ;HC CT CT HC
16 6 5 4 9 ;CT CT N4 CT
16 6 5 13 9 ;HC CT N4 CT
16 6 5 14 9 ;HC CT N4 CT
24 6 5 4 9 ;CT CT N4 H
24 6 5 13 9 ;HC CT N4 H
24 6 5 14 9 ;HC CT N4 H
44 6 5 4 9 ;CT CT N4 H
44 6 5 13 9 ;HC CT N4 H
44 6 5 14 9 ;HC CT N4 H
17 16 6 5 9 ;CT CT N4 CT
17 16 6 24 9 ;CT CT N4 H
17 16 6 44 9 ;CT CT N4 H
25 16 6 5 9 ;HC CT N4 CT
25 16 6 24 9 ;HC CT N4 H
25 16 6 44 9 ;HC CT N4 H
26 16 6 5 9 ;HC CT N4 CT
26 16 6 24 9 ;HC CT N4 H
26 16 6 44 9 ;HC CT N4 H
18 17 16 6 9 ;CT CT CT N4
18 17 16 25 9 ;CT CT CT HC
18 17 16 26 9 ;CT CT CT HC

27 17 16 6 9 ;HC CT CT N4
27 17 16 25 9 ;HC CT CT HC
27 17 16 26 9 ;HC CT CT HC
28 17 16 6 9 ;HC CT CT N4
28 17 16 25 9 ;HC CT CT HC
28 17 16 26 9 ;HC CT CT HC
19 18 17 16 9 ;CT CT CT N4
19 18 17 27 9 ;HC CT CT N4
19 18 17 28 9 ;HC CT CT N4
29 18 17 16 9 ;CT CT CT HC
29 18 17 27 9 ;HC CT CT HC
29 18 17 28 9 ;HC CT CT HC
30 18 17 16 9 ;CT CT CT HC
30 18 17 27 9 ;HC CT CT HC
30 18 17 28 9 ;HC CT CT HC
37 19 18 17 9 ;CT CT N4 H
37 19 18 29 9 ;HC CT N4 H
37 19 18 30 9 ;HC CT N4 H
38 19 18 17 9 ;CT CT N4 H
38 19 18 29 9 ;HC CT N4 H
38 19 18 30 9 ;HC CT N4 H
42 19 18 17 9 ;CT CT N4 H
42 19 18 29 9 ;HC CT N4 H
42 19 18 30 9 ;HC CT N4 H
21 20 1 2 9 ;CT CT N4 CT
21 20 1 15 9 ;CT CT N4 H
21 20 1 43 9 ;CT CT N4 H
31 20 1 2 9 ;HC CT N4 CT
31 20 1 15 9 ;HC CT N4 H
31 20 1 43 9 ;HC CT N4 H
32 20 1 2 9 ;HC CT N4 CT
32 20 1 15 9 ;HC CT N4 H
32 20 1 43 9 ;HC CT N4 H
22 21 20 1 9 ;CT CT CT N4
22 21 20 31 9 ;CT CT CT HC
22 21 20 32 9 ;CT CT CT HC
33 21 20 1 9 ;HC CT CT N4
33 21 20 31 9 ;HC CT CT HC
33 21 20 32 9 ;HC CT CT HC
34 21 20 1 9 ;HC CT CT N4
34 21 20 31 9 ;HC CT CT HC
34 21 20 32 9 ;HC CT CT HC
23 22 21 20 9 ;CT CT CT N4
23 22 21 33 9 ;HC CT CT N4
23 22 21 34 9 ;HC CT CT N4

35 22 21 20 9 ;CT CT CT HC
35 22 21 33 9 ;HC CT CT HC
35 22 21 34 9 ;HC CT CT HC
36 22 21 20 9 ;CT CT CT HC
36 22 21 33 9 ;HC CT CT HC
36 22 21 34 9 ;HC CT CT HC
39 23 22 21 9 ;CT CT N4 H
39 23 22 35 9 ;HC CT N4 H
39 23 22 36 9 ;HC CT N4 H
40 23 22 21 9 ;CT CT N4 H
40 23 22 35 9 ;HC CT N4 H
40 23 22 36 9 ;HC CT N4 H
41 23 22 21 9 ;CT CT N4 H
41 23 22 35 9 ;HC CT N4 H
41 23 22 36 9 ;HC CT N4 H

References

(1) Case, D. A.; Cheatham III, T. E.; Darden, T.; Gohlke, H.; Luo, R.; Merz Jr, K. M.; Onufriev, A.; Simmerling, C.; Wang, B.; Woods, R. J. The Amber biomolecular simulation programs. *Journal of computational chemistry* **2005**, *26*, 1668–1688.

(2) Hess, B.; Kutzner, C.; Van Der Spoel, D.; Lindahl, E. GROMACS 4: algorithms for highly efficient, load-balanced, and scalable molecular simulation. *Journal of chemical theory and computation* **2008**, *4*, 435–447.

(3) Pérez, A.; Marchán, I.; Svozil, D.; Sponer, J.; Cheatham III, T. E.; Laughton, C. A.; Orozco, M. Refinement of the AMBER force field for nucleic acids: improving the description of α/γ conformers. *Biophysical journal* **2007**, *92*, 3817–3829.

(4) Yoo, J.; Aksimentiev, A. Improved parametrization of Li+, Na+, K+, and Mg2+ ions for all-atom molecular dynamics simulations of nucleic acid systems. *The journal of physical chemistry letters* **2012**, *3*, 45–50.

(5) Jorgensen, W. L.; Chandrasekhar, J.; Madura, J. D.; Impey, R. W.; Klein, M. L. Comparison of simple potential functions for simulating liquid water. *The Journal of chemical physics* **1983**, *79*, 926–935.

(6) Yoo, J.; Aksimentiev, A. Improved parameterization of amine–carboxylate and amine–phosphate interactions for molecular dynamics simulations using the CHARMM and AMBER force fields. *Journal of chemical theory and computation* **2016**, *12*, 430–443.

(7) Bayly, C. I.; Cieplak, P.; Cornell, W.; Kollman, P. A. A well-behaved electrostatic potential based method using charge restraints for deriving atomic charges: the RESP model. *The Journal of Physical Chemistry* **1993**, *97*, 10269–10280.

(8) Yoo, J.; Kim, H.; Aksimentiev, A.; Ha, T. Direct evidence for sequence-dependent attraction between double-stranded DNA controlled by methylation. *Nature communications* **2016**, *7*, 11045.

(9) Miyamoto, S.; Kollman, P. A. Settle: An analytical version of the SHAKE and RATTLE algorithm for rigid water models. *Journal of computational chemistry* **1992**, *13*, 952–962.

(10) Hess, B.; Bekker, H.; Berendsen, H. J.; Fraaije, J. G. LINCS: a linear constraint solver for molecular simulations. *Journal of computational chemistry* **1997**, *18*, 1463–1472.

(11) Berendsen, H. J.; Postma, J. v.; van Gunsteren, W. F.; DiNola, A.; Haak, J. R. Molecular dynamics with coupling to an external bath. *The Journal of chemical physics* **1984**, *81*, 3684–3690.

(12) Parrinello, M.; Rahman, A. Polymorphic transitions in single crystals: A new molecular dynamics method. *Journal of Applied physics* **1981**, *52*, 7182–7190.

(13) Bussi, G.; Donadio, D.; Parrinello, M. Canonical sampling through velocity rescaling. *The Journal of chemical physics* **2007**, *126*, 014101.

(14) Barducci, A.; Bussi, G.; Parrinello, M. Well-tempered metadynamics: a smoothly converging and tunable free-energy method. *Physical review letters* **2008**, *100*, 020603.

(15) He, W.; Naleem, N.; Kleiman, D.; Kirmizialtin, S. Refining the RNA force field with small-angle X-ray scattering of helix–junction–helix RNA. *The Journal of Physical Chemistry Letters* **2022**, *13*, 3400–3408.

(16) He, W.; Kirmizialtin, S. Exploring Cation Mediated DNA Interactions Using Computer Simulations. International Conference on Bio and Nanomaterials. 2019; pp 51–63.

(17) Caro, M. A.; Laurila, T.; Lopez-Acevedo, O. Accurate schemes for calculation of thermodynamic properties of liquid mixtures from molecular dynamics simulations. *The Journal of chemical physics* **2016**, *145*, 244504.

(18) Lin, S.-T.; Maiti, P. K.; Goddard III, W. A. Two-phase thermodynamic model for efficient and accurate absolute entropy of water from molecular dynamics simulations. *The Journal of Physical Chemistry B* **2010**, *114*, 8191–8198.

(19) Ali, H. S.; Higham, J.; Henchman, R. H. Entropy of Simulated Liquids Using Multiscale Cell Correlation. *Entropy* **2019**, *21*, 750.

(20) Chakravorty, A.; Higham, J.; Henchman, R. H. Entropy of Proteins Using Multiscale Cell Correlation. *Journal of Chemical Information and Modeling* **2020**, *60*, 5540–5551.
